# Veterinary Medical Association of New Jersey

**Published:** 1901-02

**Authors:** 


### VETERINARY MEDICAL ASSOCIATION OF NEW JERSEY.

President Lowe announces the appointment of Committees and Delegates for the consolidated State Association of New Jersey as follows:

Executive Committee.—Dr. William Herbert Lowe, 188–190 Ellison Street, Paterson, *Chairman;* Drs. George W. Pope, Garfield; T. E. Smith, Jersey City; John B. Hopper, Ridgewood; James M. Mecray, Maple Shade; L. P. Hurley, Hopewell, and S. S. Treadwell, Englewood.

Public Health Committee.—Dr. Werner Runge, Newark, *Chairman;* Drs. G. P. Harker, Trenton; J. V. Laddey, Arlington; Whitfield Gray, Newton, and L. R. Sattler, Newark.

Animal Industry Committee.—Dr. J. Payne Lowe, Passaic, *Chairman;* Drs. R. R. Letts, Jersey City; W. F. Harrison, Bloomfield; John B. Finch, Ramsey, and James W. Hawk, Newark.

Legislation Committee.—Dr. T. Earle Budd, Orange, *Chairman;* Drs. A. G. Vogt, Newark; William B. E. Miller, Camden; T. E. Smith, Jersey City, and Henry Vander Roest, Newark.

State Committee on Atlantic City Meeting, A. V. M. A.—Dr. James T. Glennon, Newark, *Chairman;* Drs. John O. George, Camden; E. L. Loblein, New Brunswick; E. R. Voorhees, Somerville, and J. Payne Lowe, Passaic.

Finance Committee.—Dr. R. O. Hasbrouck, Passaic, *Chairman;* Drs. Ernest Buckley, Orange, and David J. Dixon, Hoboken.

Publication Committee.—Dr. George W. Pope, Garfield, *Chairman;* Drs. S. Lockwood, Woodbridge, and M. M. Stage, Dover.

Committee on Clinic for Newark Meeting.—Dr. Werner Runge, Newark, *Chairman;* Drs. James McDonough, Montclair, and E. A. Hogan, Newark.

ESSAYISTS FOR NEWARK MEETING.—Dr. George W. Pope, Garfield; Dr. L. R. Sattler, Newark, and Dr. G. P. Harker, Trenton.

DELEGATES TO THE AMERICAN VETERINARY MEDICAL ASSOCIATION.—Dr. J. M. Everitt, Hackettstown; Dr. A. W. Axford, Naughright; Dr. W. J. Fredericks, Delawanna; Dr. William Gall, Matawan, and Dr. S. S. Treadwell, Englewood.

DELEGATES TO THE PENNSYLVANIA STATE VETERINARY MEDICAL ASSOCIATION.—Dr. L. P. Hurley, Hopewell; Dr. William B. E. Miller, Camden; Dr. Edgar L. Landes, Camden; Dr. Charles E. Magill, Haddonfield, and Dr. H. W. Read, Freehold.

DELEGATES TO THE NEW YORK STATE VETERINARY MEDICAL SOCIETY.—Dr. E. Mathews, Jersey City; Dr. A. H. McIntosh, Summit; Dr. James McDonough, Montclair; Dr. James D. Hopkins, Newark, and Dr. John Kehoe, Lyndhurst.

DELEGATES TO THE NEW JERSEY STATE SANITARY ASSOCIATION.—Dr. J. V. Laddey, Arlington; Dr. James C. Corlies, Newark, and Dr. G. P. Harker, Trenton.

